# Prevalence of psychiatric and cognitive disorders in patients with Pierre-Robin sequence - a statistical analysis

**DOI:** 10.1192/j.eurpsy.2024.1007

**Published:** 2024-08-27

**Authors:** G. Pereira Bernd, V. Dall Agnol Bouvier, T. Brusa da Costa Linn, I. Cho de Almeida, B. de Oliveira de Marchi, L. Guinter Muccillo, C. G. Menezes Chaves Barcellos, C. Paz Portinho, M. V. Martins Collares

**Affiliations:** ^1^Universidade Federal do Rio Grande do Sul, Porto Alegre; ^2^Feevale, Novo Hamburgo, Brazil

## Abstract

**Introduction:**

The Pierre-Robin sequence (PRS), characterized by micrognathia, glossoptosis, and cleft palate, has long been a subject of clinical interest. Recent research suggests a potential association between PRS and cognitive or psychiatric disorders. This study explores this intriguing connection, shedding light on the complex interplay between craniofacial anomalies and mental health.

**Objectives:**

This study aims to establish a comprehensive understanding of the relationship between Pierre-Robin Sequence and psychiatric disorders. Specifically, our objectives include: assessing prevalence, evaluating impact and informing clinical practice. This research aims to improve the holistic care and mental well-being of individuals with craniofacial malformations, contributing to a more comprehensive approach in the field of psychiatry.

**Methods:**

This cross-sectional study was conducted at a prominent referral hospital named Hospital de Clínicas de Porto Alegre, an international reference in Pierre-Robin Sequence, during the month of August 2023.

Participant Selection: Patients with PRS. Inclusion criteria encompassed individuals of all ages and both genders.

Data Collection: Trained medical personnel conducted structured interviews with participants to gather demographic information, medical history, and details of their craniofacial conditions.

Medical Records Review: Medical records were reviewed to corroborate craniofacial diagnoses and identify any comorbid conditions.

Statistical Analysis: Data were analyzed using appropriate statistical techniques to assess the association between PRS and psychiatric disorders.

Ethical Considerations: The study adhered to all ethical guidelines, with informed consent obtained from participants or their legal guardians. Ethical approval was obtained from the hospital’s Institutional Review Board.

Data Handling: Confidentiality and data security were ensured throughout the study, with all data anonymized to protect participant privacy.

**Results:**

In our study, we assessed 28 different patients with Pierre-Robin Sequence, comprising 13 females and 15 males. The youngest patient was 2 months old, while the oldest was 22 years old. The mean age of the patients was 4.75 years, with a median of 3 years and a standard deviation of 5.36 years.

Among the patients, 6 exhibited psychiatric disorders, split between 4 males and 2 females. Their average age was 10 years, with a median of 9 years and a standard deviation of 4.2. The youngest patient with evidence of a psychiatric disorder was 5 years old.

**Conclusions:**

This study underscores a concerning reality within the Pierre-Robin population, pointing to a high prevalence of psychiatric disorders. These findings highlight the urgent need for integrated care, emphasizing the importance of early psychiatric assessment and tailored interventions to enhance the overall well-being of individuals facing the challenges of PRS.

**Disclosure of Interest:**

None Declared

